# The Noncoding Mutational Landscape of Pancreatic Cancer Reveals Recurrent Somatic Mutations in Enhancer Regions

**DOI:** 10.1158/2767-9764.CRC-24-0167

**Published:** 2025-10-17

**Authors:** Akimasa Hayashi, Yu-Jui Ho, Alvin P. Makohon-Moore, Amanda Zucker, Jungeui Hong, Shigeaki Umeda, Elias-Ramzey Karnoub, Jinlong Huang, Priscilla Baez, Rajya Kappagantula, Jerry P. Melchor, Wungki Park, Eileen M. O’Reilly, Nicholas D. Socci, Shinya Oki, Christine A. Iacobuzio-Donahue

**Affiliations:** 1 David M. Rubenstein Center for Pancreatic Cancer Research, Sloan Kettering Institute, Memorial Sloan Kettering Cancer Center, New York, New York.; 2 Human Oncology and Pathogenesis Program, Sloan Kettering Institute, Memorial Sloan Kettering Cancer Center, New York, New York.; 3 Department of Pathology, Kyorin University School of Medicine, Mitaka, Japan.; 4 Cancer Biology and Genetics Program, Sloan Kettering Institute, Memorial Sloan Kettering Cancer Center, New York, New York.; 5 Center for Discovery and Innovation, Hackensack Meridian Health, Nutley, New Jersey.; 6 Georgetown University Lombardi Comprehensive Cancer Center, Washington, District of Columbia.; 7 Gastrointestinal Oncology Service, Division of Solid Tumor Oncology, Department of Medicine, Memorial Sloan Kettering Cancer Center, New York, New York.; 8 Weill Cornell Department of Medicine, Weill Cornell Medicine, New York, New York.; 9 Bioinformatics Core, Sloan Kettering Institute, Memorial Sloan Kettering Cancer Center, New York, New York.; 10 Institute of Resource Development and Analysis, Kumamoto University, Kumamoto, Japan.

## Abstract

While the coding genome of pancreatic cancer has been characterized in detail, features of the noncoding genome remain relatively unexplored. We used whole-genome sequencing to study the coding and noncoding genomes (promoters, enhancers, and noncoding–nonenhancer regions) in two unique patient cohorts. We find that treated cancers have a significantly higher mutational burden than untreated cancers in all four genomic regions. However, the relative proportion of mutations in each region is preserved despite treatment-induced genetic bottlenecks. Compared with other noncoding regions, enhancers have a lower number of mutations/Mb. Enhancers also have a distinct mutational signature with enrichment of SBS39. Enhancer sequences were segregated into conserved and nonconserved regions based on the overlap of predicted orthologous regions in the human and mouse genomes and the conserved regions screened for recurrent somatic mutations. We find that recurrent somatic mutations in conserved enhancer regions largely correspond to those associated with known transcription factors with a role in pancreatic development and cancer, including KLF5 and TP63. Transcriptional expression based on RNA sequencing data of cancers with enhancer mutations showed significantly different levels of expression, most often a loss of expression, compared with cancers without enhancer mutations, suggesting a functional effect. These findings expand our knowledge of the noncoding genome in pancreatic cancer and point to an unexplored role of conserved enhancer mutations for pancreatic cancer as well.

**Significance:**

Recurrent somatic mutations in enhancer regions that control pancreatic lineage genes represent a previously unrecognized source of genetic alteration in pancreatic cancer.

## Introduction

Despite the wealth of data pertaining to the genetics of pancreatic ductal adenocarcinoma (PDAC), the full spectrum of genetic features of this tumor type have yet to be discovered (1, 2). To date, large-scale whole-exome sequencing studies have revealed the recurrent genomic features of PDAC that target a defined number of core pathways (3–7). High-frequency driver gene mutations in PDAC include *KRAS*, *TP53*, *CDKN2A*, and *SMAD4*, which play a major role in clonal expansions during pancreatic carcinogenesis and influence metastatic propensity. Subsequent subclonal genetic events are also key for tumor progression and metastasis, for example copy number gains of *cMYC* or *GATA6* (8, 9).

Emerging data indicate that the noncoding genome also plays a role in cancer. For example, increased long interspersed element 1 activity has been associated with multiple tumor types in which it may contribute to somatic structural variations in known driver genes (10). A comprehensive reanalysis of 2,583 whole genomes from 27 tumor types by the Pan-Cancer Analysis of Whole Genomes consortium has also identified several novel recurrent somatic alterations of the noncoding genome (11). These include recurrent microdeletions in *BRD4*, point mutations in the 3′ regions of *NFKBIZ* and *TOB1*, point mutations in the 5′ untranslated regions and promoters of *TP53* and *MTG2*, and structural rearrangements at chromosome 10p15 affecting the *AKR1C* locus (11). In PDAC specifically, Feigin and colleagues (12) found an impact of mutations in promoter regions, specifically cis-regulatory regions of genes associated with transcriptional regulation. Fewer studies have focused specifically on enhancer mutations (13, 14), although there is accumulating evidence that mutations in enhancer regions may play an important role in transcriptional changes through transcription factor dynamics (15, 16). In some tumor types such as bladder (17) and breast cancers (18), enhancer mutations are thought to be bona fide driver mutations, an observation also supported by pan-cancer analyses (19, 20).

Collectively, whereas the noncoding genome seems to play a role in cancer in general, these alterations are much less frequent than those affecting the coding genome (11). Moreover, tissue specificity plays a role in identification of recurrent noncoding somatic alterations (11); hence, reports of noncoding alterations in pan-cancer studies may underrepresent those alterations specific to given tumor type. Even less well understood is the timing of accumulation of noncoding alterations with respect to the clonal progression of solid tumors (21). To address this gap in knowledge for PDAC, we performed multiregional whole-genome sequencing (WGS) and clonal analysis to elucidate the characteristic features of noncoding regions relative to those of the coding genome, including their evolutionary relationships to other known PDAC features.

## Materials and Methods

### Ethics statement

Written informed consent was obtained from all patients whose tissues were used. The study was conducted in accordance with recognized ethical guidelines (e.g., Declaration of Helsinki, CIOMS, Belmont Report, and US Common Rule) and approved by the institutional review boards of the Johns Hopkins School of Medicine and Memorial Sloan Kettering Cancer Center (MSKCC).

All animal studies conducted at MSKCC were approved by the Institutional Animal Care and Use Committee.

### Tissue samples

Samples from six PDAC research autopsies (MPAM01-06) from the Last Wish Program at MSKCC were used (Supplementary Table S1). Sections were cut from frozen sections and reviewed to identify those with at least 20% neoplastic cellularity and preserved tissue quality. Normal samples were reviewed to confirm that no contaminating cancer cells were present. Samples meeting these criteria were macrodissected to enrich for tumor purity from serial unstained sections before extraction of genomic DNA using QIAamp DNA Mini Kit (Qiagen). Four treatment-naïve PDAC cases previously analyzed through the Rapid Autopsy Program at Johns Hopkins University were also included in this study (Supplementary Table S1; ref. 22).

### WGS

Raw data from WGS for PAM01-04 were previously generated (22). Genomic DNA was extracted from all tissues for patients MPAM01-06. DNA quantification, library preparation, and WGS were performed in the MSK Integrated Genomics Operation, and bioinformatics analysis of somatic variants was performed by the MSK Bioinformatics Core. Sequencing, alignment, and analysis of MPAM01-06 were performed as described for PAM01-04 (22). Briefly, Illumina HiSeq 2000, HiSeq 2500, HiSeq 4000, or NovaSeq 6000 platform was used to target a coverage of 60X or 80X for tumor and 60X or 30X for normal samples. The resulting sequencing reads were analyzed *in silico* to assess quality, coverage, as well as alignment to the human reference genome using Burrows–Wheeler Aligner (23). After read deduplication, base quality recalibration, and multiple sequence realignment were completed with the Picard suite and GATK version 3.1 (24, 25), somatic single-nucleotide variants **(**SNV)/insertions–deletions were detected using MuTect version 1.1.6 and HaplotypeCaller version 2.4 (24, 25). We excluded low-quality or poorly aligned reads from phylogenetic analysis (24, 26). A median coverage of 76.5× (range, 59–124) in tumor and 47.5× (range, 28–76) in normal was obtained throughout this cohort (Supplementary Table S2).

### Filtering and annotation of variants

For all patients, somatic variants were filtered using the following criteria: patient-matched normal coverage ≥10 reads, variant count in patient-matched normal ≤2, patient-matched normal variant frequency <0.02, tumor mutant ≥10 reads, and tumor variant allele frequency ≥0.05 in at least one tumor sample, under condition with tumor coverage ≥20 reads in all samples. Coding variants were subject to further bioinformatic annotation for pathogenicity and germline allele frequencies from healthy populations distributed worldwide using LiFD (Supplementary Table S3; ref. 27). Copy number alterations and whole genome duplication were inferred by FACETs (28).

### RNA sequencing

RNA extraction and sequencing was performed as recently described (8). Briefly total RNA was extracted using TRIzol (Life Technologies) followed by RNeasy Plus Mini Kit (Qiagen). After library preparation using the TruSeq Stranded Total RNA LT Kit (Illumina, catalog # RS-122-1202), samples were barcoded and run on a HiSeq 4000 in a 100 bp/100 bp or 125/125 bp paired-end run using the HiSeq 3000/4000 SBS Kit (Illumina). Output data (FASTQ files) were mapped to the target genome using the rnaStar aligner (version 2.5.0a; ref. 29), and postprocessing of the output SAM files was performed using PICARD tools to add read groups and covert it to a compressed BAM format. The expression count matrix from the mapped reads was determined using HTSeq (https://htseq.readthedocs.io/en/release_0.11.1), and the raw count matrix generated by HTSeq was processed using the R/Bioconductor package DESeq2 (http://bioconductor.org/packages/release/bioc/html/DESeq2.html) to normalize the entire dataset between sample groups. Log_2_-transformed data were used as normalized expression for downstream analyses.

### The Cancer Genome Atlas data set

The Cancer Genome Atlas (TCGA) pancreas cancer WGS data were obtained from Data Coordinating Center Data Releases/International Cancer Genome Consortium (ICGC) Data Portal (https://dcc.icgc.org/releases/release_27/Projects/PACA-CA). Mutation calls were obtained from MuTect, and a total of 2,271,144 unique mutations were identified. These mutations were reannotated with vcf2maf tool and new annotated files as input for downstream analysis.

TCGA pancreatic cancer (version 2016_01_28 for Pancreatic adenocarcinoma) RNA sequencing (RNA-seq) data were downloaded through FireBrowse (http://firebrowse.org). Transcripts per million (TPM) was calculated from downloaded RNA-seq data. TPM was used for gene set enrichment analysis, and log-2 converted TPM values were used as relative mRNA expression.

### Pancreatic cancer enhancer regions and enhancer mutations

Enhancer regions were previously defined based on H3K27ac (activated enhancer histone marks) chromatin immunoprecipitation sequencing (ChIP-seq) data (30, 31). Twenty-three H3K27ac ChIP datasets of pancreatic cancer were selected from ChIP atlas (https://chip-atlas.org/) based on number of peaks (>10,000) in combination with manual review on Integrated Genomics Viewer (Supplementary Table S4). All data were merged and analyzed using MACS2 (32) and identifying regions with scores more than 50 [−10 × log_10_ (MACS2 *Q*-value)]. These regions were annotated for the closest and second closest genes using GREAT (version 4.0.4; ref. 33). Regions located between 2 and 50 kb from the transcription start site were included, whereas those farther than 50 kb or closer than 2 kb from the transcription start site were excluded. Overall, 62,015 regions were included as “pancreatic cancer enhancer regions” in this study (Supplementary Data). Enhancer mutations were defined as somatic variants in enhancer regions that overlapped with PDAC enhancer regions using Galaxy bedtools ClosestBed (https://usegalaxy.org/; version 19.09; ref. 34). Representative enchanter mutations were confirmed with Droplet Digital PCR (ddPCR) according to the previous study (35).

### Mutational signature analysis

Palimpsest (https://github.com/FunGeST/Palimpsest) was used for mutational signature analysis (34). All mutations identified in each sample in coding, enhancer, promoter, or noncoding–nonenhancer regions (hereafter referred to as noncoding-NOS) were used as inputs. Signature proportions in each category of each sample were used for clustering analysis as input to identify characteristics of enhancer region mutational signatures. Wilcoxon signed-rank test was performed to identify characteristic signatures between groups. Mutational signature *t* test with FDR approach using the *t*-stage set-up method of Benjamini, Krieger, and Yekutieli was performed to identify characteristic signatures between clonal versus subclonal and treated and untreated groups.

### Evolutionary analysis

We derived phylogenies for each set of samples by using Treeomics 1.7.9 (36). Each phylogeny was rooted at the matched patient’s normal sample, and the leaves represented tumor samples. Treeomics uses a Bayesian inference model to account for error-prone sequencing and varying neoplastic cell content to calculate the probability that a specific variant is present or absent. The global optimal tree is based on mixed-integer linear programming. All evolutionary analyses were performed based on WGS. Somatic alterations present in all analyzed samples of a PDAC were considered clonal, whereas those in a subset of samples or in a single sample were considered subclonal.

### Conserved pancreatic cancer enhancer regions and gene annotation

Conserved enhancer regions between humans and mouse were obtained from a previous ENCODE study (37). Pancreatic cancer enhancer regions with any overlap with ENCODE conserved enhancer regions were defined as “conserved pancreatic cancer enhancer regions.” Of 62,015 pancreatic cancer enhancer regions, 8,966 regions (14.5%) passed this criteria. Each conserved enhancer region and mutation included in these regions (i.e., conserved enhancer mutations) was assigned to the first and second nearest genes within 500 kb using GREAT (version 4.0.4; ref. 33).

### Conserved enhancer mutation and gene expression

Gene expression was compared between samples with and without conserved enhancer mutations using previously generated RNA-seq data for the autopsy cohort (8) and ICGC cohort (38). Samples with nonsynonymous coding mutations were removed from this analysis to avoid potential effects on transcript stability. Changes in gene expression per gene were compared using a Wilcoxon signed-rank test. To confirm the reliability of this analysis, for each gene, the mutation categories were randomly shuffled across samples, using conserved pancreatic cancer enhancer regions as a reference. Expression levels were compared using the Wilcox test. This procedure was repeated 10,000 times to generate a distribution of reference *P* values. The original *P* value was then compared with these reference values, and the proportion of reference *P* values less than the original was calculated, resulting in an updated adjusted *P* value.

Genes with significant upregulated or downregulated changes in autopsy and/or TCGA cohort were used for Gene Ontology analysis with Enrichr (https://amp.pharm.mssm.edu/Enrichr/; ref. 39).

### Allele-specific expression analysis

Allele-specific expression (ASE) analysis was performed using cis-X, following the methodology described in the original publication (40). Briefly, we organized multimodal genomic data from our PDAC samples for integrated analysis using the cis-X computational framework. Input data included copy number variation profiles generated by Control-FREEC, SNVs, and insertions/deletions called by Strelka2, RNA expression quantified as FPKM values using STAR and featureCounts, and WGS alignment files. The cis-X Singularity container with default parameters was used for the ASE analysis.

### Motif analysis on enhancer sequences

The original mutation summary table (from autopsy and the ICGC cohort) was used to extract genes showing significant expression differences between mutations in conserved pancreatic cancer enhancer regions and wild-type variants. Mutation locations were then extracted from the raw mutation data and mapped to the original conserved enhancer regions described above. These conserved enhancer regions were analyzed using findGenomeMotif.pl from Hypergeometric Optimization of Motif EnRichment to identify known motifs in each region and the extent to which mutations occurred within these motifs.

### Statistics and reproducibility

All statistics and graphs were performed and made using GraphPad Prism (version 8.2.1) and/or R (version 3.6.1). Parametric distributions were compared by a two-sided *χ*^2^ test, with correction using a Fisher exact test for sample sizes <5. Nonparametric distributions were compared using a Mann–Whitney U test (two-sided) and, for analysis of contingency tables, two-sided Fisher exact test was used. Each analysis is described in the Results section. Overall survival analyses were performed using the Kaplan–Meier method, and curves were compared by a log-rank test. Statistical significance was considered if *P* value is less than 0.05. FDR *q*-value was used for gene set enrichment analysis.

No statistical method was used to predetermine sample size. No data were excluded from the analyses as long as the library and/or sequencing quality passed our criteria. The experiments were not randomized. The investigators were not blinded to allocation during experiments and outcome assessment except histologic slide review.

## Results

### Clinicopathologic and genetic features of the patient cohort

**Figure 1.**
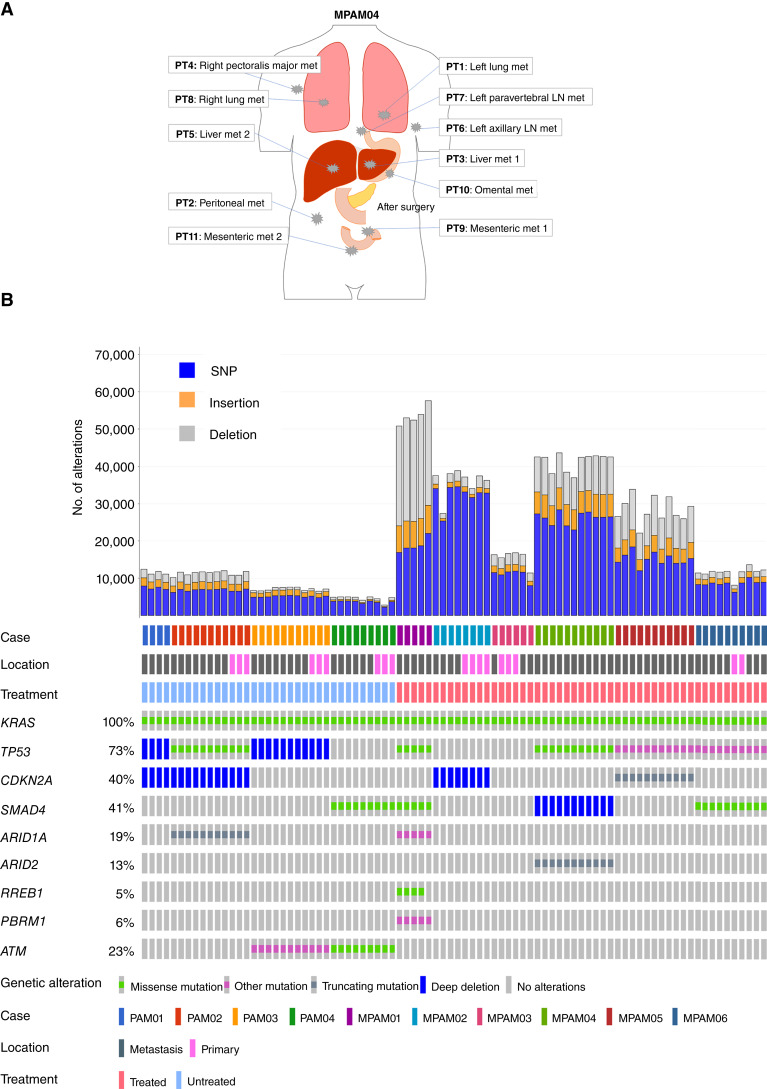
Mutational overview of end-stage PDACs in the autopsy cohort. **A,** Schematic of multisampling in the representative autopsy case (MPAM04). One normal tissue from each patient was also sequenced to aid identification of somatic variants. **B,** Summary of the number of somatic mutations (histogram) and the coding driver gene alterations (oncoprint) identified in each sample. Patients PAM01–PAM04 were treatment-naïve and MPAM01–MPAM06 were treated with chemotherapy with or without radiation. LN, lymph node; met, metastasis; PT, patient tumor sample.

The clinical features of this cohort are summarized in Supplementary Table S3 and Supplementary Fig. S1. Eight of 10 patients are diagnosed at clinical stage IV pancreas cancer, and two are stage IIB (surgically resectable). The latter two patients underwent a pancreaticoduodenectomy with curative intent but subsequently developed recurrent metastatic disease. Four patients (PAM01-04) were untreated due to poor performance status at diagnosis and had a short survival (range, 0.5–10 months), whereas six patients (MPAM01-06) had chemotherapy and a relatively longer survival (range, 9–49 months). At autopsy, all 10 patients had pathologically confirmed metastases in at least one organ (Fig. 1A). Nine of 10 cases were ductal adenocarcinoma, and one case (MPAM02) had features of an undifferentiated carcinoma with osteoclast-like giant cells. Two cases of ductal adenocarcinoma, PAM02 and MPAM06, showed focal squamous differentiation, and a third case (PAM01) had neuroendocrine features.

A total of 86 PDAC genomes and 10 normal genomes from these 10 patients (median 10 samples per patient; range, 4–11) were used for this study (Supplementary Table S4). We identified a median of 15,312 somatic mutations per sample (range, 2,972–58,657) corresponding to median values of 11,671 (range, 2,329–37,225) SNVs, 1,903 (213–7,889) small insertions, and 2,664 (430–28,224) small deletions per sample (Fig. 1B). Coding driver gene mutations or copy number changes of *KRAS* (10/10, 100%), *TP53* (7/10, 73%), *CDKN2A* (4/10, 40%), *SMAD4* (4/10, 40%), *ATM* (2/10, 20%), *ARID1A* (2/10, 10%), *ARID2* (1/10, 10%), and *PBRM1* (1/10, 10%) were detected, consistent with findings of previous large cohort studies (Fig. 1B; refs. 6, 41). To understand the relationship between treatment and mutational load, we independently evaluated cases with respect to clinical history of receiving standard-of-care chemotherapy. Untreated PDACs had a significantly lower number of mutations (median 7,543 mutations per sample; range, 2,783–12,415 vs. median 31,914 per sample, 8,172–57,585; *P* < 0.0001, Mann–Whitney U test, two-sided) than treated PDACs.

**Figure 2.**
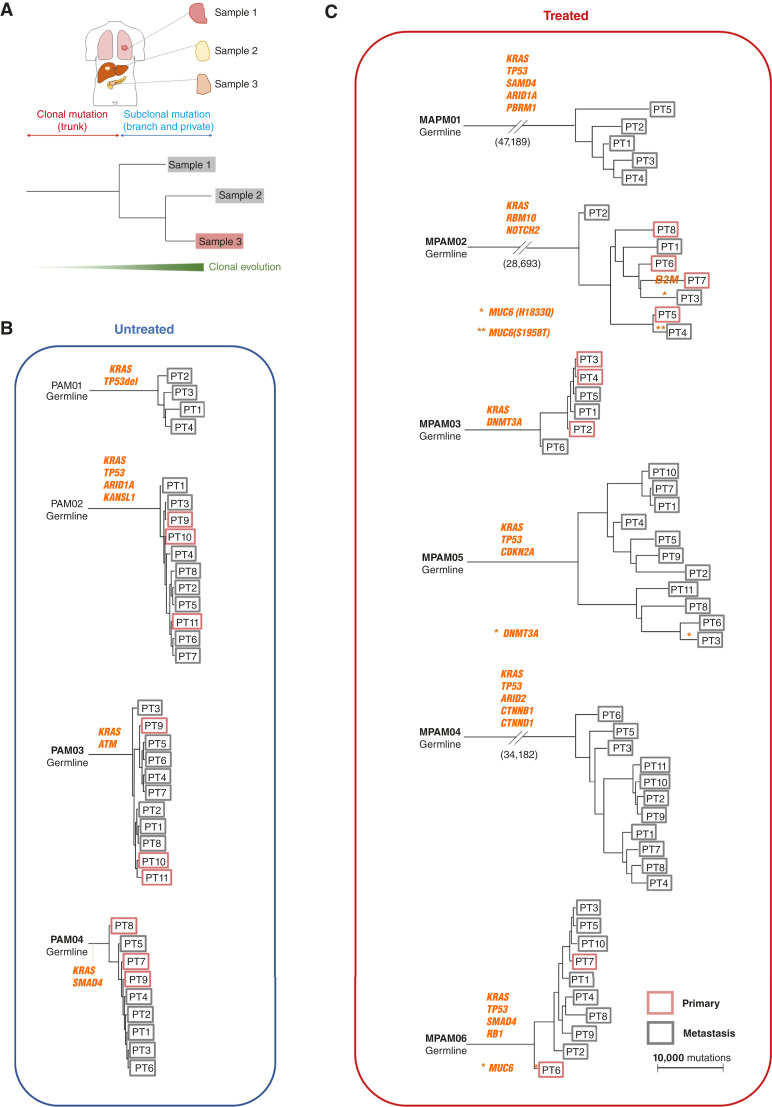
Phylogenies of end-stage PDAC. (**A**) Schematics of the clonal and subclonal mutations in phylogenies. (**B** and **C**) Phylogenies of end (**B**) untreated and (**C**) treated cases of end-stage PDAC. PT, patient. * and ** indicate the presence of driver genes at the same branch of each case phylogeny. The specific gene names are shown in the corresponding case phylogenies.

Sample phylogenies were derived for each patient to determine the relative evolutionary timing of somatic variant occurrence. Common driver genes were truncal in origin in all patients (Fig. 2A–C). Three PDACs, all from treated patients (MPAM02, MPAM05, and MAPM06), had subclonal driver gene mutations in *MUC6*, *B2M*, or *DNMT3A* (Fig. 2C; Supplementary Table S3). Each of these subclonal driver gene alterations was private to a single sample analyzed from the patient, most often within the primary tumor, suggestive of treatment-induced genetic bottlenecks (35, 42). Consistent with this interpretation, treated PDACs have longer branches compared with untreated PDACs (combination of external and internal branches; both *P* < 0.0001; Mann–Whitney U test, two-sided). In six of 10 cases, at least one primary tumor sample was most closely related to a metastasis, indicative of subclonal heterogeneity and formation of metastatic subclones within the primary tumor, as previously described (8, 22, 35). Collectively, these findings demonstrate that this PDAC cohort exhibits the expected clonal dynamics for driver genes and provides the baseline for interpretation of noncoding genetic alterations in advanced PDAC.

### Mutational characteristics of the PDAC genome

**Figure 3.**
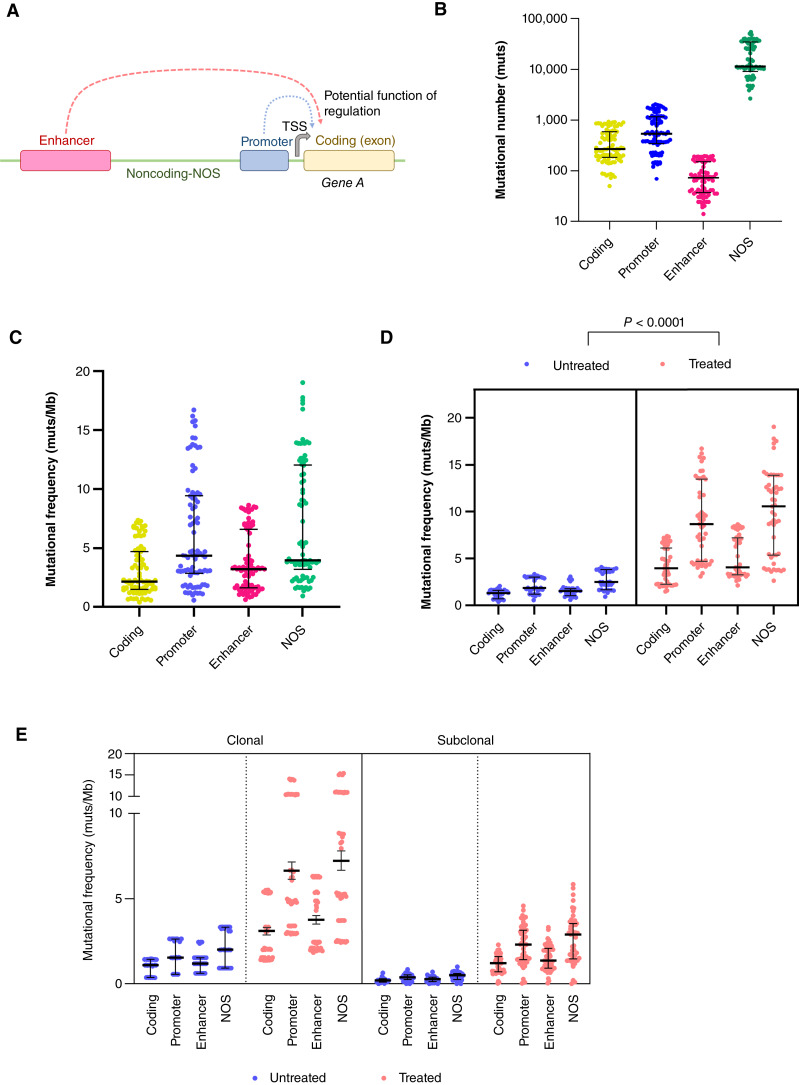
Mutational frequency in PDAC enhancers. **A,** Schematic of coding, promoter, enhancer, and noncoding-NOS regions. **B,** Overall mutational number and (**C**) mutational frequency per Mb in coding, promoter, enhancer, and noncoding-NOS regions. **D,** Mutational frequency in coding, promoter, and enhancer regions in untreated and treated PDAC. **E,** Clonal and subclonal mutational frequency in coding, promoter, and enhancer regions in untreated and treated PDAC. TSS, transcription start site.

All mutations were subclassified into those occurring in coding, promoters, enhancers, or noncoding-NOS regions (Fig. 3A; Supplementary Table S5). Overall, the fewest mutations were present in enhancer regions (median of 73 mutations, range, 14–198), and the most in noncoding-NOS regions (median of 11,312 mutations, range, 2,651–54,532; Fig. 3B). When we considered the mutational frequency per million bases (mut/Mb), we find a median of 2.13 (range, 0.38–7.37), 4.38 (0.56–16.71), 3.19 (0.61–8.64), and 3.94 (0.93–19.03) mutations in coding, promoter, enhancer, and noncoding-NOS regions, respectively (Fig. 3C; see “Materials and Methods”). To determine the extent to which treated PDACs differ from untreated PDACs, we calculated the mut/Mb in each of the genomic regions in each group (Fig. 3D). In untreated PDACs, we find median values of 1.30 coding (range, 0.39–2.06), 1.86 promoter (range, 0.56–3.33), 1.52 enhancer (range, 0.61–3.05), and 2.48 noncoding-NOS (range, 0.93–4.07) mut/Mb. In treated PDACs, the median mut/Mb per region were at least three times higher, with values of 3.95 in coding (range, 1.49–7.37), 8.67 in promoter (range, 3.10–16.71), 4.05 in enhancer (range, 2.14–8.64), and 10.56 (range, 2.63–19.03) in noncoding-NOS regions (Fig. 3D). Pairwise comparisons of the mutational frequency in treated PDACs compared with untreated indicates that the mutational burden is significantly greater in all regions of the genome compared with untreated PDAC (*P* < 0.0001, Mann–Whitney test).

We next determined the extent to which the relative timing of accumulation of mutations occurs during the evolutionary life history of each PDAC (Fig. 3E). In untreated PDACs, we find median values of 1.10 (range, 0.35–1.45), 1.54 (range, 0.55–2.63), 1.18 (range, 0.61–2.44), and 2.00 (range, 0.91–3.35) clonal mut/Mb and 0.20 (range, 0–0.64), 0.38 (range, 0–0.84), 0.26 (range, 0–0.70), and 0.50 (range, 0.003–0.99) subclonal mut/Mb in coding, promoter, enhancer, and noncoding NOS regions, respectively (Fig. 3E), indicating that the majority of mutations occur prior to formation of the most recent common ancestor. We find a similar relationship in treated PDACs, with median values of 2.05 (range, 1.37–5.52), 4.91 (range, 2.89–14.07), 2.44 (range, 1.83–6.33), and 5.31 (range, 2.41–15.38) clonal mut/Mb and 1.21 (range, 0.03–2.29), 2.30 (range, 0.03–4.58), 1.35 (range, 0.04–3.32), and 2.90 (range, 0.01–5.84) subclonal mut/Mb in coding, promoter, enhancer, and noncoding NOS regions, respectively. Collectively, these findings suggest two features of the noncoding genome: first, most mutations occur prior to formation of the most recent common ancestor, and second, the proportion of mutations in coding, promoter, enhancer, and noncoding-NOS regions is preserved despite treatment-induced genetic bottlenecks.

### Mutational signatures in PDAC

**Figure 4.**
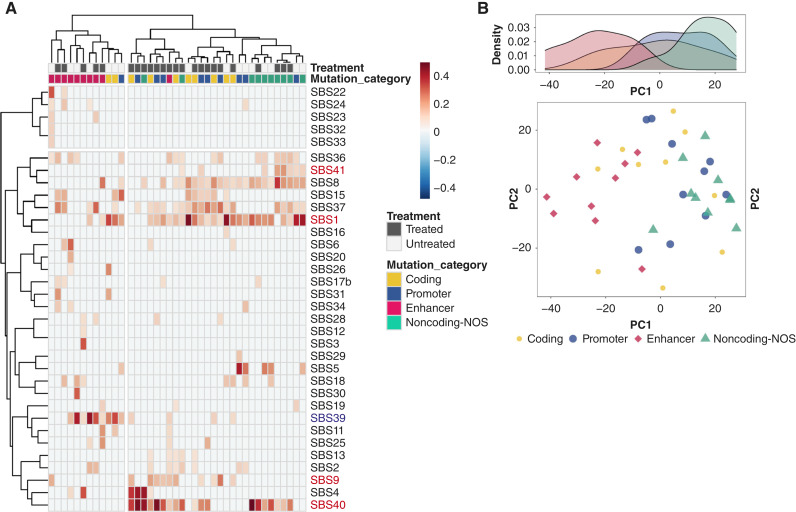
Mutational signature in PDAC enhancers. **A,** Heatmap of mutational signature in coding, promoter, enhancer, and noncoding-NOS regions. **B,** Principal component analysis based on mutational signature.

To identify the general characteristics of mutational signatures in each genomic region, we used Palimpsest (34) and created a heatmap based on mutational proportions for each cohort (Fig. 4A; Supplementary Fig. S2A). Unsupervised clustering analysis based on combined mutational signatures identified two groups within which the four genomic regions were nonrandomly distributed. Specifically, enhancer regions exhibit distinct signature characteristics compared with other regions of the genome (Fig. 4A). This is accompanied by significant enrichment of the SBS39 signature (*P* = 0.0029, Friedman test) and reduction of the SBS1 (*P* < 0.001, Friedman test), SBS8 (*P* < 0.001), SBS40 (*P* < 0.001), and SBS41 (*P* < 0.001) signatures in enhancer regions (Supplementary Tables S6 and S7). To account for sample size effect (i.e., alteration counts of noncoding-NOS >> coding ≈ enhancer), we performed subsampling analysis using the identical number of mutations (coding counts) for all categories for each case and confirmed that the clustering results are similar. Principal component (PC) analysis based on mutational signatures (Fig. 4B) also identified enhancer alterations as distinct in the first PC (PC1).

We next determined the extent to which these mutational signatures correlate with other features of PDAC such as clonality of mutations or treatment. SBS1, a clock-like mutational signature, and SBS18, which is associated with reactive oxygen species (43), were the only two signatures that were more prevalent in the clonal fraction of mutations relative to those that arise during subclonal evolution (Supplementary Fig. S2A; Supplementary Table S8). Enhancer alterations also remained distinct in the PC1 when subclassified as clonal or subclonal (Supplementary Fig. S2B). SBS1 and SBS5 (also an age-related signature) are also significantly more prevalent in untreated cases (Wilcoxon signed-rank test *P* value = 0.019 and 0.026; Supplementary Table S9), consistent with enrichment for alternative mutational processes during treatment or due to treatment specifically (35).

Overall, these findings indicate that mutational processes differ among these four genomic regions in a context-dependent manner, with enhancer mutations having the most distinctive signatures compared with other regions.

### Orthogonal validation in the ICGC dataset

To determine the extent to which the findings in our modest sized autopsy cohort are generalizable, we assessed mutational burdens and signatures in an orthogonal set of 259 PDACs from the ICGC (Supplementary Fig. S1; ref. 6). We identified a median of 5,899 somatic mutations per sample (range, 1,040–94,812) corresponding to median values of 5,376 (range, 887–91,013) SNVs, 270 (range, 2–10,814) insertions, and 225 (range, 11–28,315) deletions per sample (Supplementary Fig. S3A). When these mutations were classified into those occurring in coding regions, promoters, enhancers, or noncoding-NOS regions (see “Materials and Methods”), a median of 63 (range, 8–1,636), 189 (38–3,653), 27 (4–656), and 5,618 (988–89,569) mutations were identified per region, respectively. The mut/Mb revealed a median of 0.50 (range, 0.064–13), 1.54 (0.31–29.7), 1.18 (range, 0.18–28.6), and 1.96 (0.35–31.3) mutations in coding regions, promoters, enhancers, and noncoding-NOS regions, respectively (Supplementary Fig. S3B). PC analysis based on mutational signatures (Supplementary Fig. S3C) also identified enhancer alterations as distinct in PC1, although to a lesser extent than seen in the autopsy cohort. Finally, we determined the mutational signatures that are most discriminative of each genomic region. Similar to the autopsy cohort, we find that SBS39 is enriched, whereas SBS1, SBS5, SBS8, and SBS40 are underrepresented in enhancer regions (Supplementary Fig. S3D). Overall, we conclude that genomic features of the noncoding genome this large cohort of early-stage PDACs are similar to those observed in the autopsy cohort.

### Mutations in conserved enhancer regions in PDAC

**Figure 5.**
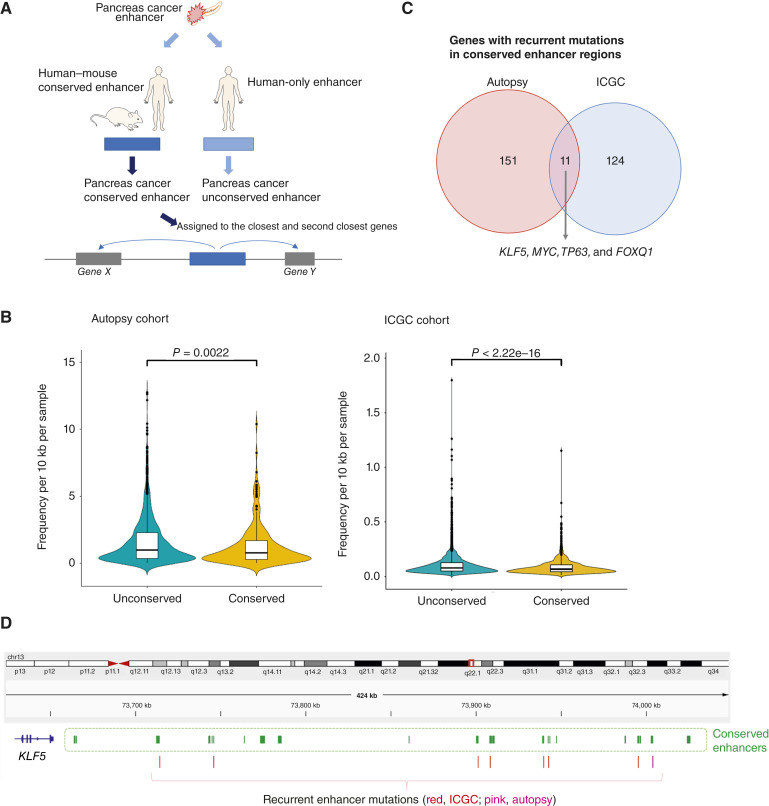
Conserved enhancer mutations and gene expression. **A,** Schematic of approach to identify conserved enhancers in the human genome. **B,** Mutational frequency in conserved and unconserved regions in the autopsy and ICGC cohorts. **C,** Genes with recurrent mutations in conserved enhancer regions. **D,** Recurrent mutations in conserved enhancers around *KLF5*.

Recurrent mutations in promoter regions have previously been reported (12). For that reason, and because of the unique features noted for enhancers in this study, we focused on the extent that enhancer mutations are recurrent in PDAC. Enhancer regions are, from the perspective of species evolution, divided into two categories: conserved and unconserved regions (37). Therefore, we relied upon previously generated ChIP-seq data that defined PDAC enhancer regions based on H3K27 activated histone marks (see “Materials and Methods”; refs. 30, 31) and classified these regions into conserved and unconserved categories based on the overlap of predicted orthologous enhancers in the mouse and human genomes (Fig. 5A; ref. 37). This strategy identified 8,966 conserved enhancer regions (Supplementary Table S10). The mutational frequency in conserved enhancer regions was lower than that in unconserved enhancer regions in both the ICGC and autopsy cohorts (Fig. 5B).

We annotated these conserved enhancer regions to identify their closest and second closest genes. Among these 5,618 genes (Fig. 5A; Supplementary Table S10), we focused on those with recurrent mutations in their associated conserved enhancer regions. In the autopsy cohort, 162 genes were mutated in three or more unique samples, 32 genes of which were affected in two or more patients (Fig. 5C; Supplementary Table S11). Representative conserved enhancer mutations were confirmed and validated by ddPCR (Supplementary Fig. S4). Eight enhancer mutations detected by WGS were confirmed using ddPCR. In the ICGC cohort, we found 135 genes for which three or more patients were mutated, 18 of which were recurrently mutated in five or more patients (Fig. 5C; Supplementary Table S11). Eleven genes were identified in both cohorts using these thresholds (three autopsy samples and three ICGC patients), several of which have been associated with cancer biology or PDAC specifically (Fig. 5C and D; refs. 44–46). These 11 genes encode for protein products that function as transcription factors or cotranscription factors (*MYC*, *KLF5*, *FOXQ1*, *POU5F1B*, *TP63*, and *LPP*), response to hypoxia (*EGLN3*; refs. 47, 48), regulation of the extracellular matrix (*HAS2*; ref. 49), and actin remodeling (*SEMA3E*; ref. 50). Genes such as *DCBLD2* (discoidin, CUB, and LCCL domain-containing 2; ref. 51) and *TPRG1* (tumor protein P63 regulated 1; ref. 52) are less well studied in cancer, although the latter (*TP63* regulated gene 1) further implicates the TP63 axis.

### Gene expression in association with mutated enhancers

Somatic alterations in conserved enhancer regions do not indicate whether changes in gene expression may occur. Therefore, transcriptional expression based on RNA-seq was evaluated for the two genes closest to each enhancer. Specifically, for each conserved enhancer with a mutation, we compared gene expression of associated genes in the same tissue (see “Materials and Methods”). For subsequent analyses, only WGS–RNA-seq matched samples were considered in both the autopsy and ICGC cohorts.

**Figure 6.**
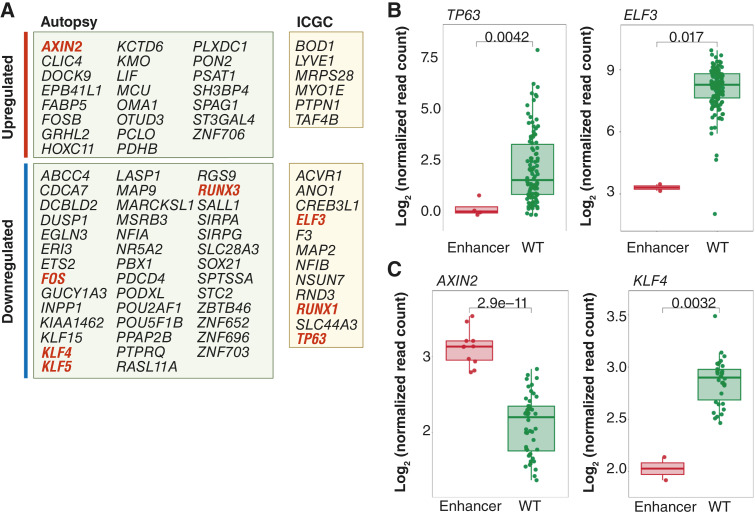
Genes with altered expression in association with conserved enhancer region mutations. **A,** Upregulated and downregulated gene list in autopsy series and the ICGC cohort. Potential driver genes are shown in bold red. **B,** Representative changes in gene expression in TP63 and ELF3 in the ICGC cohort. **C,** Representative changes in gene expression in AXIN2 and KLF4 in the autopsy cohort.

We identified 92 genes among the combined autopsy and ICGC cohorts with a significant difference in gene expression between samples with and without an enhancer mutation (Supplementary Tables S12 and S13; Fig. 6A). Gene expression was most often decreased in samples with an associated enhancer mutation (Supplementary Tables S12 and S13). Downregulated genes again included the transcription factors KLF5 and POU5F1B in the autopsy cohort or ELF3 and RUNX1 within the ICGC cohort, among many others Supplementary Tables S12 and S13). TP63 was also again identified and showed >10-fold lower gene expression in association with the enhancer mutation (Fig. 6B). By contrast, AXIN2 was notable for being increased in expression in association with somatic mutations of the nearby enhancer (Fig. 6C).

While individual genes provide some clues to the phenotypes targeted by enhancer mutations, we combined the data pertaining to significantly altered expression in association with an enhancer mutation in both the ICGC and autopsy cohorts and performed gene ontology analysis for further insight. Downregulated genes (Fig. 6A) were highly enriched for those involved in regulation of DNA-templated transcription (Supplementary Table S14). By contrast, there were no pathways identified associated with upregulated gene expression (Supplementary Table S15).

To determine the extent to which the recurrent enhancer mutations identified affect known or predicted enhancer motif regions, we used Hypergeometric Optimization of Motif EnRichment to identify regulatory elements that are specifically enriched for or against in mutated conserved enhancer regions relative to wild-type sequences (“Materials and Methods”; ref. 53). Enhancer sequences were categorized based on mutation counts (using a cutoff of three mutations to define high vs. low) and changes in gene expression (upregulated vs. downregulated). None of the genes identified by the prior analyses were identified. However, this analysis revealed two known consensus regions associated with ELF5 and Isl1 as significantly enriched (*P* < 0.0001 and *P* < 0.001, hypergeometric test; Supplementary Table S16; Supplementary Fig. S5A). We next performed a *de novo* analysis to evaluate predicted motif regions, revealing motif sequences associated with Smad2, Nanog, and Sox18 (Supplementary Fig. S5B).

## Discussion

Recent large-scale efforts have greatly contributed to the discovery and annotation of noncoding drivers of cancer in general (11) or for specific tumor types, including PDAC (12, 17, 18). We now extend the spectrum of genetic events that occur in PDAC to recurrent mutations in conserved enhancer regions. None of the genes identified in this current study were identified by the Pan-Cancer Analysis of Whole Genomes analysis of noncoding DNA, consistent with the authors’ conclusion that tumor type–specific studies will reveal additional alterations related to the cellular origin of that tissue (11, 54). In keeping with this prediction, we identified recurrent enhancer mutations with potential links to genes whose protein products contribute to pancreatic biology and homeostasis, for example *KLF5*, among many others (45, 55). Additional potential targets were identified by motif analysis such as Smad2, a canonical mediator of TGFΒ signaling. Although additional work will be required to fully understand the extent to which recurrent enhancer mutations contribute to PDAC biology, that gene expression varies significantly in association with many of these enhancer mutations, whereas others are associated with predicted regulatory motif regions, supports their having a functional role.

One finding of particular significance was recurrent enhancer mutations affecting both *TP63* and its target gene *TPRG1* in both PDAC cohorts studied. TP63 gene expression was also significantly lower in association with these mutations in the ICGC cohort, whereas TPRG1 has been identified as a recurrent target of somatic mutations in cis-regulatory regions in B-cell lymphoma (56). TP63 is a known master regulator of enhancer reprogramming, and its nuclear expression in PDAC is highly associated with basal-like or squamous features (4). We noted that two patients in our autopsy cohort had basal-like features (8), yet neither of these PDACs, or the specific region with this histology, were found to have the mutations in the TP63 nearby enhancer. Thus, it is conceivable that mutations in these regulatory elements may negatively regulate TP63 expression and reinforce classic-type PDAC phenotypes, particularly as GATA6 overexpression or amplification is an imperfect marker of classic phenotypes and response to gemcitabine/nab-paclitaxel (57). At the very least, interrogation of this conserved enhancer region in PDACs may expand the list of predictive biomarkers to first line chemotherapy for this disease (58).

The overall mutation rates found in this study are consistent with the literature and also highlight the expected effects of treatment in that treated PDACs had 3.8-fold overall higher mut/Mb than untreated PDACs. However, we now find that mutation rates differ by genomic region. Specifically, we find that the mutation rate of enhancers is significantly lower than that of other noncoding regions as well as having a distinct mutational signature. These differences likely correspond to a number of factors, including but not limited to differences in chromatin structure between enhancers (euchromatin) and intronic DNA (heterochromatin; refs. 59, 60) and differences in DNA repair efficiency among different regions of the genome (59, 61). It is conceivable that the low mutation rates found within enhancer regions may also reflect a relative negative selection compared with other noncoding regions of the genome. Traditionally, inference of positive or negative selection takes into account the rates of nonsynonymous to synonymous variants in protein coding genes (62). However, not only is this approach inadequate for noncoding DNA but it also does not account for variability in local mutation rates (63). In light of data showing that mutations within enhancer DNase I–hypersensitive sites were less common than the same sequences found in intronic regions (14), our findings most likely reflect a lower overall rate of mutation accumulation than negative selection, although dedicated studies would be needed to formally prove this likelihood.

Our study nonetheless has limitations. For example, while the autopsy cohort allowed for assessment of enhancer mutations in end-stage PDAC, it contained a small number of patients. We supplemented this with the large cohort of patients from the ICGC dataset, although RNA-seq data were not available for all patients in this latter cohort to best determine effects of conserved enhancer mutations on gene expression. We also acknowledge that enhancers may affect genes much farther away than the nearest two genes that we focused on in this study; for this reason, our findings are likely a relatively conservative representation of genes affected by enhancer mutations. Finally, although we observed some overlap between the two cohorts, most genes associated with recurrent enhancer mutations were unique to either the ICGC or autopsy cohort. To further evaluate functionality, we performed ASE analysis using cis-X in the autopsy cohort, which identified a different set of candidate genes (Supplementary Table S17). This is unsurprising, as our study focused on conserved enhancer mutations, whereas cis-X was designed to detect allele-specific effects across all enhancers. These complementary approaches naturally yield distinct candidates, together offering a broader view of enhancer-mediated regulation. Long-read sequencing to phase enhancer mutations with transcript alleles would be valuable but lies beyond the scope of this study and is noted as a limitation for future work. In summary, these findings reveal an additional layer of genomic alteration in PDAC worthy of additional studies and broaden our understanding of enhancer-mediated regulation in cancer.

## Supplementary Material

Supplementary Table 1 Clinicopathological Information of PDAC Autopsy Cases

Supplementary Table 2 Sequencing Information of PDAC Autopsy Cases

Supplementary Table 3 Driver Genes Identified by LiFD

Supplementary Table 4 Public H3K7ac ChIP-seq Data Used in This Study

Supplementary Table 5 Enhancer Mutations Identified in the Autopsy Cohort

Supplementary Table 6 Mutational Signatures of Four Genomic Regions in the Autopsy Cohort

Supplementary Table 7 Mutatioal Signatures of Coding, Enhancer and Noncoding-Nonenchaner Mutations in the ICGC Cohort

Supplementary Table 8 Mutational Signatures of Clonal and Subclonal Mutations in the Autopsy Cohort

Supplementary Table 9 Mutational Signatures of Untreated and Treated PDAC Mutations in the Autopsy Cohort

Supplementary Table 10 Closeset and Second Closest Genes of Concserved PDAC Enhancers

Supplementary Table 11 Sample (and Patient) Number with Conserved Enhancer Mutation

Supplementary Table 12 Association between Conserved Enhancer Mutations and Gene Expression in the Autopsy Cohort

Supplementary Table 13 Association between Conserved Enhancer Muations and Gene Expression in the ICGC Cohort

Supplementary Table 14 Gene Ontology Analysis with Down-Regualted Genes by Conserved Enhancer Mations

Supplementary Table 15 Gene Ontology Analysis with Up-Regualted Genes by Conserved Enhancer Mations

Supplementary Table 16 Homer Motif Enrichment Analysis of Enhancers with Mutation (Known Motifs)

Supplementary Table 17 Allele-specific expression analysis with Cis-X

Supplementary Figure 1 Study Cohorts Used in This Study.

Supplementary Figure 2 Clonal and Subclonal Mutational Signature in PDAC Enhancers.

Supplementary Figure 3 Mutational Frequency and Signature in PDAC Enhancers of ICGC cohort.

Supplementary Figure 4 Validation of Enhancer Mutations by Droplet Digital PCR.

Supplementary Figure 5 Homer Motif Enrichment Analysis of Enhancers with Mutation.

Supplementary Data Supplementary Data: Enhancer List

## Data Availability

DNA and RNA sequence data for this study have been deposited at the European Genome–phenome Archive (EGA) under accession number EGAC00001000588. The other data generated in this study are available upon request from the corresponding author.
