# Supplementary figures and images for: The Noncoding Mutational Landscape of Pancreatic Cancer Reveals Recurrent Somatic Mutations in Enhancer Regions
Source: Cancer Res Commun. 2025 Oct 17;5(10):1839–51. doi: 10.1158/2767-9764.CRC-24-0167 (PMC12531768; doi:10.1158/2767-9764.CRC-24-0167)

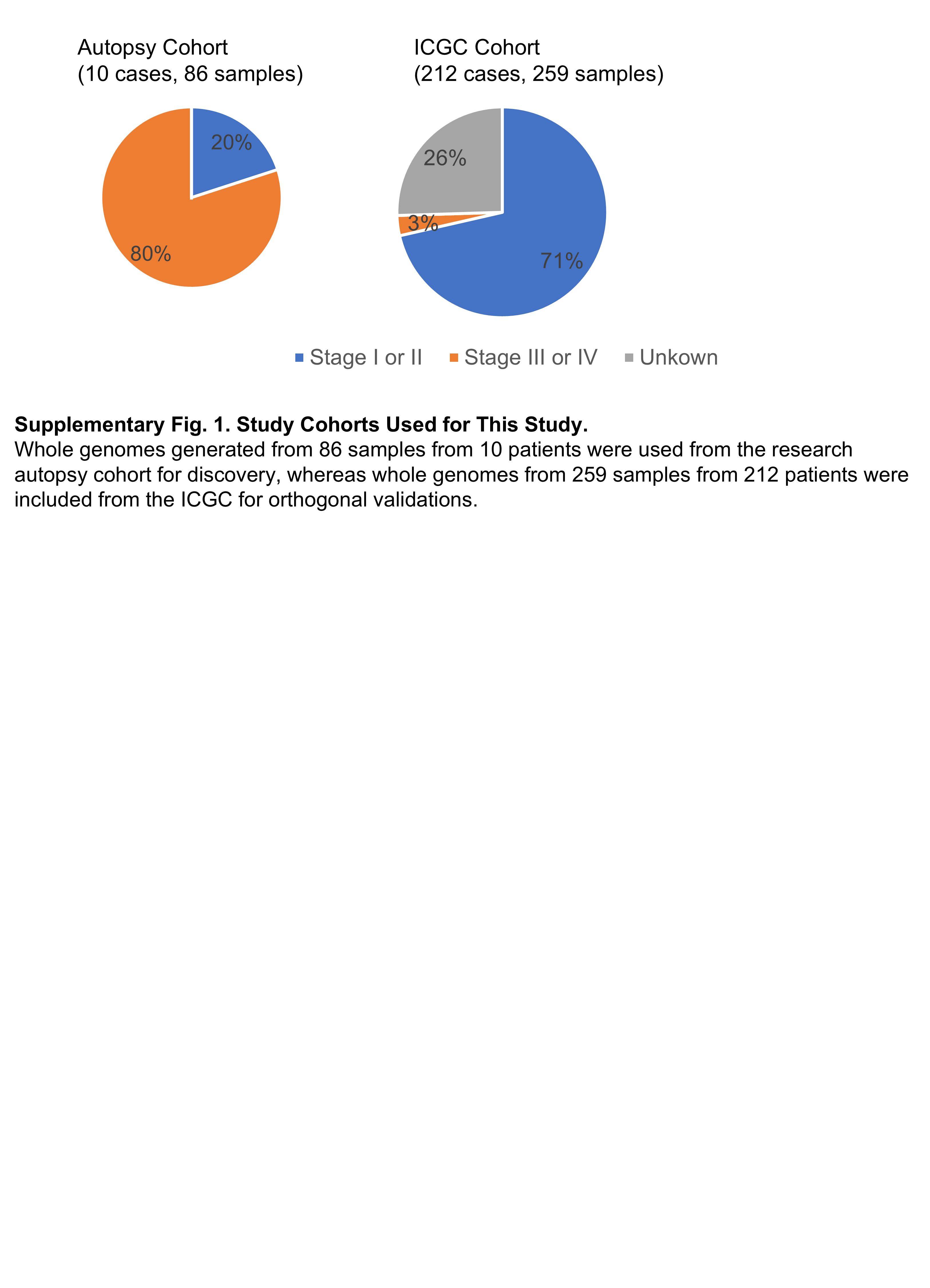

Supplement: Supplementary Figure 1 — Study Cohorts Used in This Study. [file crc-24-0167_supplementary_figure_1_suppsf1.png]

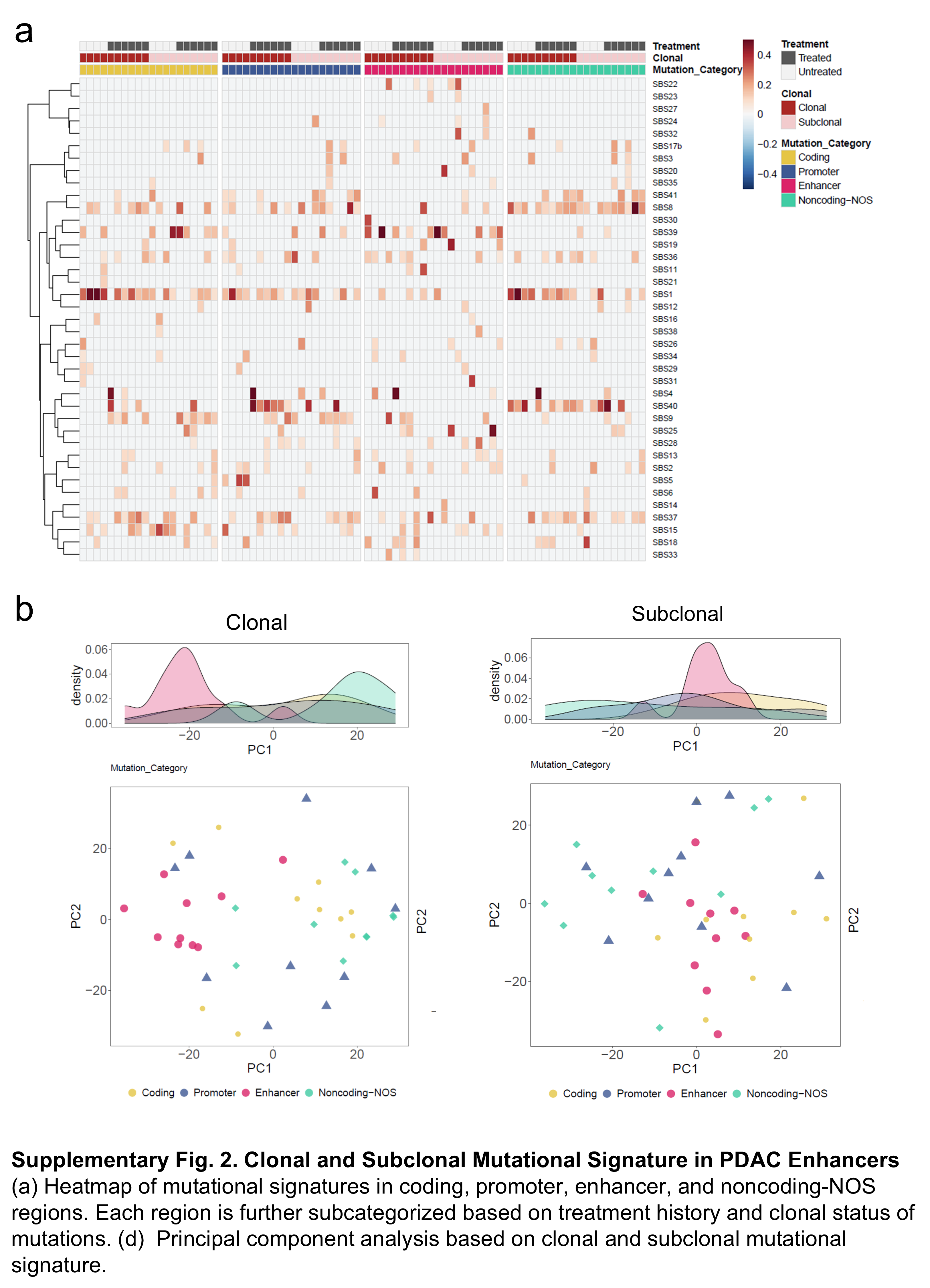

Supplement: Supplementary Figure 2 — Clonal and Subclonal Mutational Signature in PDAC Enhancers. [file crc-24-0167_supplementary_figure_2_suppsf2.png]

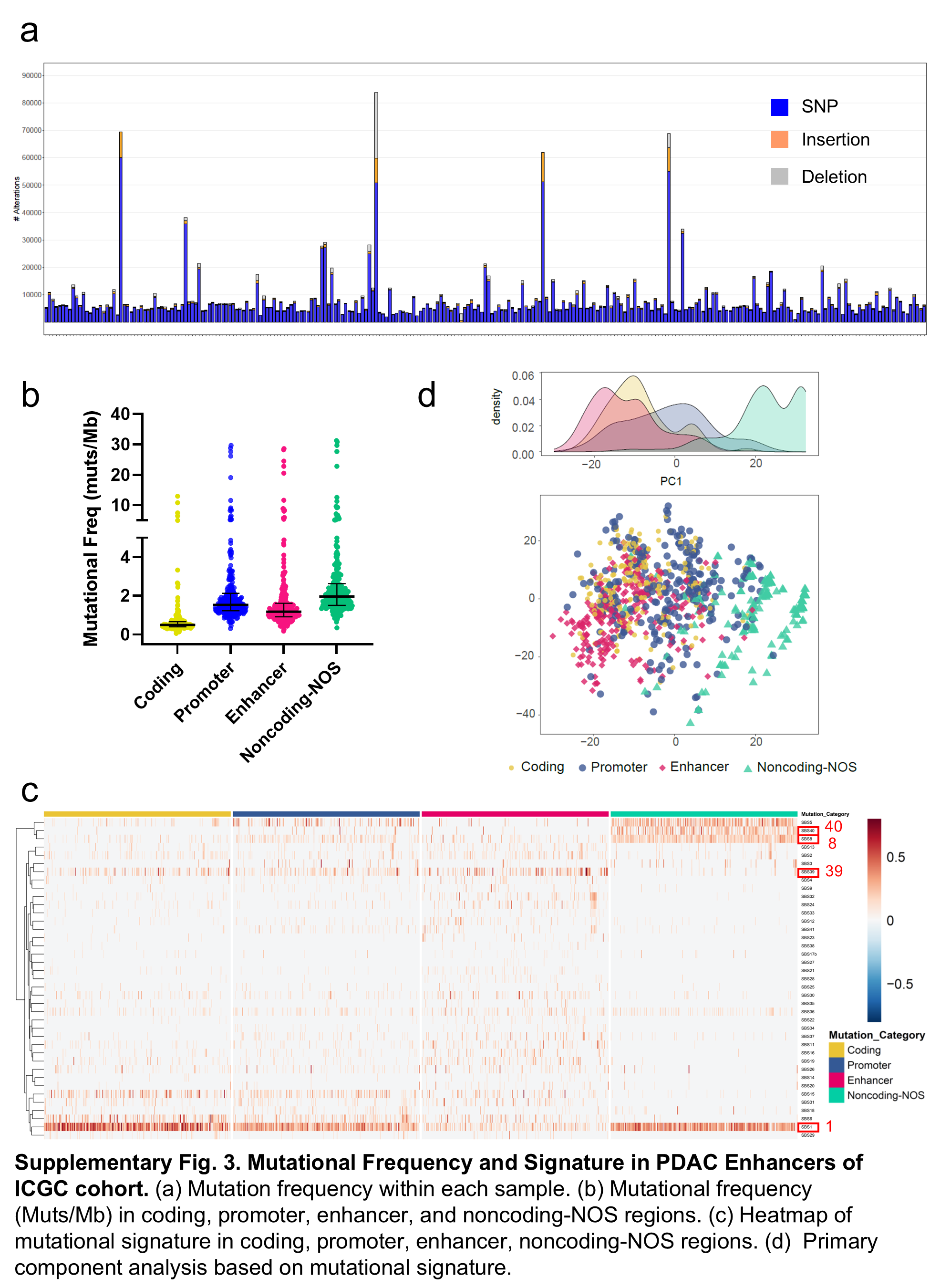

Supplement: Supplementary Figure 3 — Mutational Frequency and Signature in PDAC Enhancers of ICGC cohort. [file crc-24-0167_supplementary_figure_3_suppsf3.png]

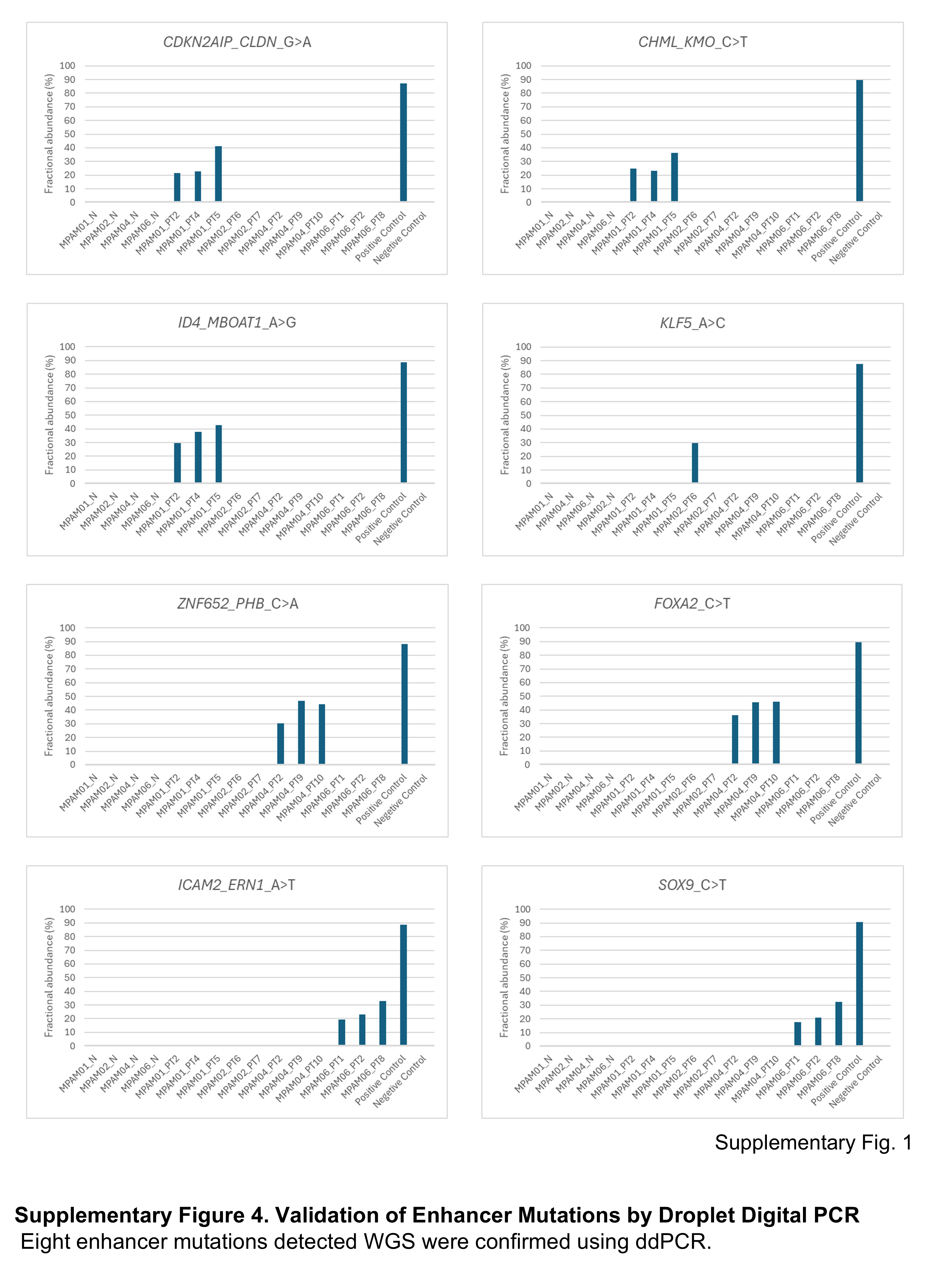

Supplement: Supplementary Figure 4 — Validation of Enhancer Mutations by Droplet Digital PCR. [file crc-24-0167_supplementary_figure_4_suppsf4.png]

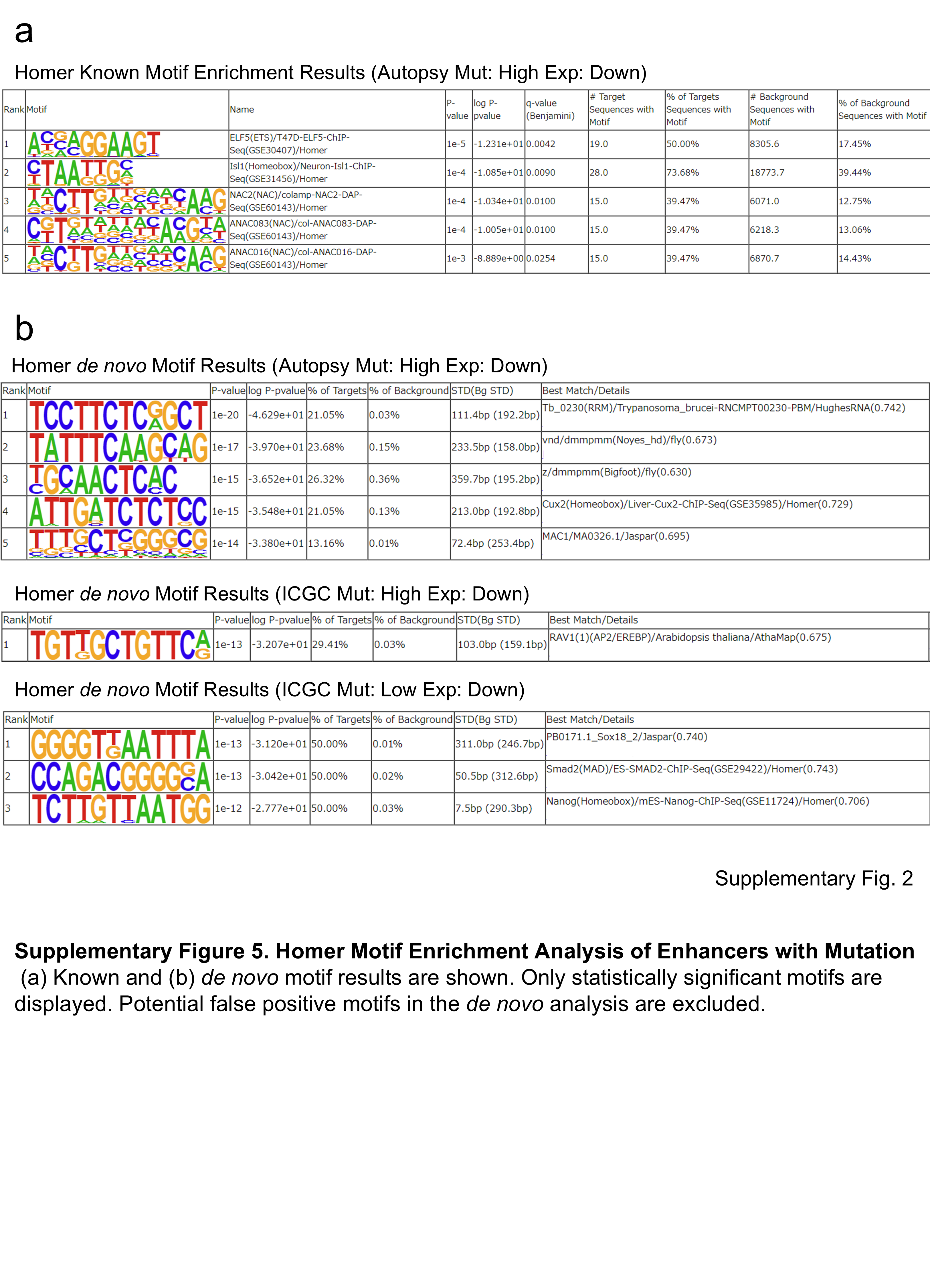

Supplement: Supplementary Figure 5 — Homer Motif Enrichment Analysis of Enhancers with Mutation. [file crc-24-0167_supplementary_figure_5_suppsf5.png]
